# Pt-free MoS_2_ co-catalyst enables record photocurrent density in Sb_2_Se_3_ photocathodes for highly efficient solar hydrogen production†

**DOI:** 10.1039/d5sc01663k

**Published:** 2025-04-16

**Authors:** Munir Ahmad, Anadil Gul, Hafiz Sartaj Aziz, Tahir Imran, Muhammad Ishaq, Muhammad Abbas, Zhenghua Su, Shuo Chen

**Affiliations:** a Institute of Thin Film Physics and Applications, Shenzhen Key Laboratory of Advanced Thin Films and Applications, Key Laboratory of Optoelectronic Devices and Systems of Ministry of Education and Guangdong Province, State Key Laboratory of Radio Frequency Heterogeneous Integration, College of Physics and Optoelectronic Engineering, Shenzhen University Shenzhen 518060 China chensh@szu.edu.cn; b College of Health Science and Environmental Engineering, Shenzhen Technology University Shenzhen 518118 China

## Abstract

Antimony selenide (Sb_2_Se_3_) emerges as a potential light-absorbing material for thin film photovoltaics and photoelectrochemical (PEC) water-splitting devices, due to its earth-abundant constituents and excellent photoelectric properties. However, losses caused by corrosion and sluggish charge transfer at the semiconductor/electrolyte interface require a co-catalyst to enhance these kinetic factors. In this study, MoS_2_ is employed as a cost-effective, noble-metal-free catalyst to enhance the photocurrent density (*J*_ph_), half-cell solar-to-hydrogen (HC-STH) conversion efficiency and stability of Sb_2_Se_3_-based photocathodes. Optimized thermodynamic/kinetic physical vapor deposition of MoS_2_ substantially improves PEC performance, resulting champion Mo/Sb_2_Se_3_/CdS/MoS_2_ photocathode that achieves a record *J*_ph_ of 31.03 mA cm^−2^ at 0 *V*_RHE_ and the highest HC-STH efficiency of 3.08%, along with stability for over 5 hours in an acidic (pH 1) buffer solution. It is systematically revealed that MoS_2_ reduces the photo-corrosion effect, decreases electron–hole recombination, and provides a significant increase in charge transfer efficiency at the semiconductor/electrolyte interface. This work highlights the potential of cost-effective, high-performance Sb_2_Se_3_-based photocathodes in advancing efficient PEC devices for solar hydrogen production.

## Abstract 2

MoS_2_ serves as low-cost and Pt-free co-catalyst that improves charge carrier dynamics in Mo/Sb_2_Se_3_/CdS/MoS_2_ photocathode. Its optimized integration minimizes interfacial recombination, leading to record *J*_ph_ of 31.03 mA cm^−2^ and HC-STH of 3.08%.

## Graphical abstract

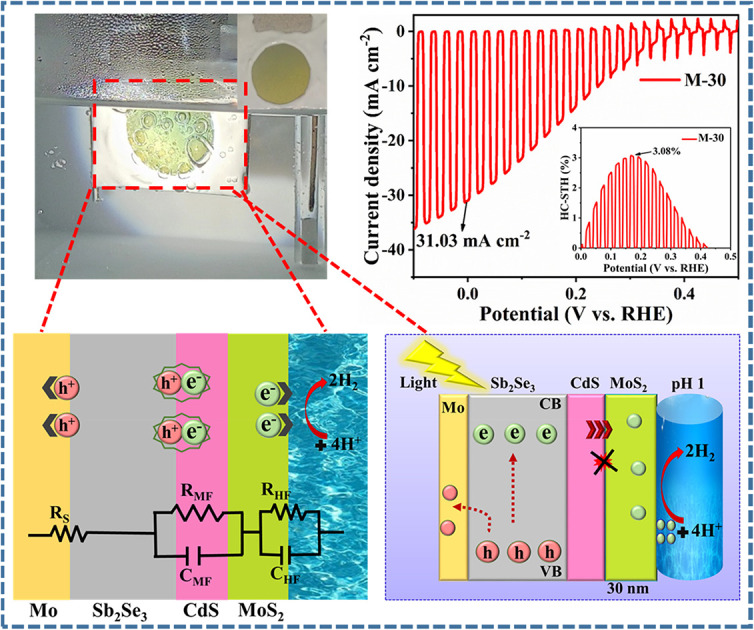

## 1. Introduction

Hydrogen production by solar-driven water splitting in photoelectrochemical (PEC) devices is one of the most attractive strategies to convert clean and abundant radiation into non-polluting and storable chemical fuels.^1^ Hydrogen energy is a carbon-free fuel that can be used in various industries, transportation, and heating applications. Currently, burning fossil fuels produces the majority of hydrogen, which leads to serious carbon emission problems.^2^ Production of green hydrogen energy through sunlight harvesting is a highly efficient approach for overcoming worldwide environmental problems and energy shortages; however, it remains challenging.^3^ Recently, different research groups have focused on introducing new photocatalysts or advanced methods to produce hydrogen energy through water splitting. Among these technologies, PEC solar water splitting is a clean, sustainable, and cost-effective, technique to produce hydrogen from solar irradiation.^4^ Some well-studied oxides, for example, CuFeO_2_^5^ and Cu_2_O,^6^ and different chalcogenides like CuInS_2_,^7^ CuGaSe_2_,^8^ and CuIn_1−*x*_Ga_*x*_Se_2_ (ref. 9 and 10) as well as newly emerging materials like Cu_2_ZnSn(S,Se)_4_,^2^ Cu_2_BaSn(S,Se)_4_ ^11^ and Sb_2_Se_3_  ^12–14^ are studied as photocathode materials.

Among the materials for hydrogen production, Sb_2_Se_3_ has emerged as an excellent light-absorbing material for PEC applications because of its earth-abundant elements, low-cost, eco-friendly, high absorption coefficient (>10^5^ cm^−1^), suitable bandgap (∼1.2 eV), and remarkable photoelectric properties. Additionally, Sb_2_Se_3_ exhibits intrinsic stability in neutral and acidic electrolytes, with negligible self-reduction or photo-corrosion, making it a suitable material for efficient PEC photocathodes.^15^ Despite these advantages, Sb_2_Se_3_-based photocathodes face many challenges such as surface corrosion caused by multiple functional layers, electron–hole non-radiative recombination caused by defects, and sluggish charge transfer at the semiconductor/electrolyte interface, which limit their PEC efficiency and long-term stability. To address these challenges, employing a suitable co-catalyst on the photocathode surface is very important. Noble metals like platinum (Pt) have been widely used as co-catalysts in Sb_2_Se_3_-based photocathodes, but their high cost and rare availability limit their commercial applications.^16,17^ Hence, the search for cost-effective and non-toxic alternative co-catalysts is highly desirable for developing efficient Sb_2_Se_3_-based photocathodes.

Recently, the development of co-catalysts like metal alloys, carbides, phosphides, nitrides, borides, and chalcogenides for the representative hydrogen evolution reaction (HER) has made significant progress.^18,19^ Among them, molybdenum sulfide (MoS_2_) has gained attention as a promising noble-metal-free co-catalyst. As a transition metal dichalcogenide (TMD), MoS_2_ possesses a suitable 2D layered structure, excellent chemical stability, and favorable band alignment for inducing the HER.^20,21^ Additionally, amorphous MoS_2_ has a high concentration of active sites at the edge layers, which enhances its electrocatalytic activity as compared to the crystalline form.^22^ A solution-based approach to synthesize MoS_2_ (crystalline/amorphous) has been used in different photocathodes (*e.g.*, Cu_2_O, Cu_2_ZnSnS_4_, and Sb_2_Se_3_).^23,24^ Actually, it still encounters challenges in terms of scalability, thickness uniformity, and composition variability.^24^ In contrast, physical vapor deposition (PVD) techniques, such as sputtering, provide a suitable preparation method for producing uniform, large-scale, and stable MoS_2_ thin film co-catalysts; however, this approach remains underexplored.^25^

This work started the preparation of eco-friendly Sb_2_Se_3_ light-harvesting films with favorable growth orientation and large crystal grains through an efficient combination reaction involving pre-sputtered and post-selenized Sb metallic precursors. After CdS buffer layer deposition, the binary compound MoS_2_ was sputtered as a co-catalyst instead of using noble-metal Pt, with a focus on MoS_2_ co-catalyst thickness engineering to enhance both light-harvesting efficiency and HER activity. In short, the introduction of optimized MoS_2_ could significantly alleviate the photo-corrosion effect, suppress charge carrier recombination loss, and reduce the charge transfer resistance at the semiconductor/electrolyte interface. Moreover, the MoS_2_ incorporation contributed to a favorable surface wettability with more reaction sites and favorable surface band bending with accelerated photoelectron transfer characteristics. As a result, the champion MoS_2_-modified Pt-free Sb_2_Se_3_ photocathode exhibited a record photocurrent density (*J*_ph_) of 31.03 mA cm^−2^ at 0 V *versus* the reversible hydrogen electrode (RHE, *V*_RHE_), and the highest half-cell solar-to-hydrogen (HC-STH) conversion efficiency of 3.08% in a pH 1 acid buffer solution. Furthermore, the device exhibited a significant improvement in long-term stability as compared to its Pt-involved counterpart. This work highlights the potential of PVD-processed MoS_2_ as a cost-effective and highly-efficient co-catalyst, advancing the development of efficient and stable Sb_2_Se_3_-based photocathodes for solar hydrogen production.

## 2. Experimental section

### 2.1. Preparation of Sb_2_Se_3_/CdS/MoS_2_ photocathodes

The Sb_2_Se_3_ light-absorbing layer and CdS buffer layer were sequentially deposited on a Mo-coated soda lime glass substrate according to our previously reported work.^26^ After the deposition of Sb_2_Se_3_ and CdS, the MoS_2_ layer with different thicknesses ranging from 20 to 40 nm was deposited as a co-catalyst by RF (radio frequency) magnetron sputtering using a MoS_2_ target. It was carried out with a 50 W sputtering power, an Ar gas flow of 50 sccm, and a working pressure of 2 Pa. The MoS_2_ layer thickness was controlled by sputtering deposition times of 400 s, 600 s, and 800 s, yielding 20, 30, and 40 nm MoS_2_ layers based on a 0.05 nm s^−1^ deposition rate, and the corresponding samples were labelled as M-20, M-30, and M-40 respectively. Finally, the Mo layer was exposed at the thin film edge to achieve a conductive back contact, and Ag colloids were deposited on its surface by thermal evaporation. The schematic of the synthesis process of glass/Mo/Sb_2_Se_3_/CdS/MoS_2_ photocathode is shown in Fig. S1 (ESI).†

### 2.2. Characterization

The crystallinity of Sb_2_Se_3_-based photocathodes was studied by X-ray diffraction (XRD) using an Ultima-IV diffractometer with Cu K_α_ radiation. The morphologies of the surface, cross-section, and structure were acquired using a scanning electron microscope (SEM, Zeiss SUPRA 55) and the corresponding elemental composition was studied by using an energy dispersive spectroscope (EDS, BRUKER QUANTAX 200). The valence states of the involved elements were studied using X-ray photoelectron spectroscopy (XPS, Thermo Scientific ESCALAB 250Xi). *In situ* sputter etching was performed using a 10 kV Gas Cluster Ion Beam (GCIB) with a 6 × 6 mm^2^ surface treatment area during the XPS measurement. Atomic force microscopy (AFM) operated, *via* NT-MDT spectrum instruments in semi-contact mode, was used for the investigation of thin film morphology and surface roughness. Surface potential and topography characterization were further analyzed through a Kelvin probe force microscope (KPFM, Bruker Dimension ICON). Raman spectroscopy (Renishaw, InVia) was used for studying bonding information. PEC performance characterization was performed with an electrochemical workstation (CHI660e) under a three-electrode configuration, *i.e.*, Ag/AgCl electrode as the reference electrode, Pt-wire as the counter electrode, and the as-fabricated photocathode as the working electrode. All tests were performed in acid electrolyte under simulated sunlight illumination (AM 1.5G) at a calibrated light intensity of 100 mW cm^−2^. Photoelectrochemical impedance spectroscopy (PEIS) was performed under simulated sunlight illumination in a 10^−1^ to 10^2^ kHz frequency range at 0 *V*_RHE_. Mott–Schottky (M–S) experiments were performed in the dark using a 30 mV AC amplitude, scanning voltage between −0.5 and 0.1 V and a frequency of 10^4^ Hz.

## 3. Results and discussion

**Fig. 1:**
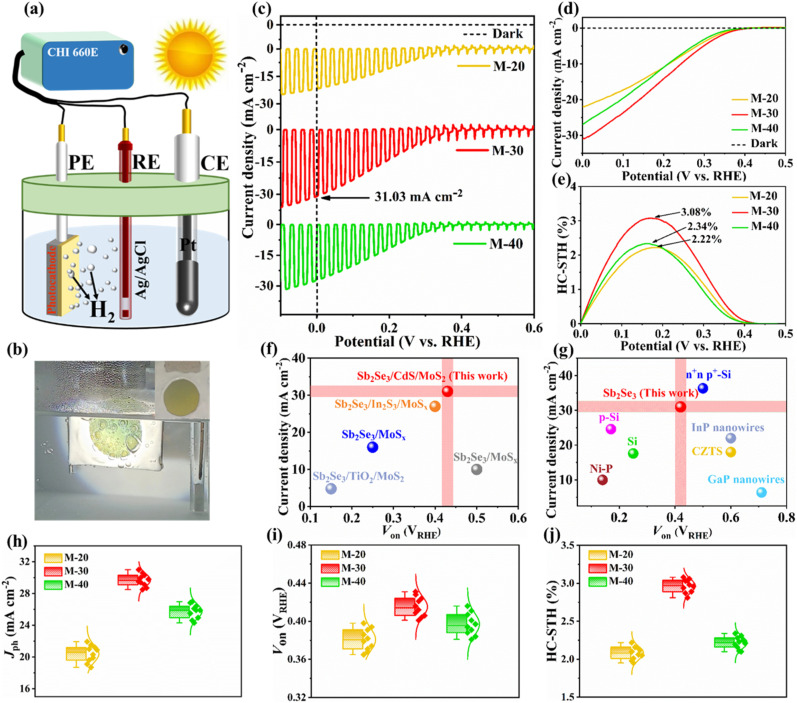
(a) Schematic diagram of a typical three-electrode PEC measurement system. (b) A picture of hydrogen bubbles originating from the surface of the photocathode and moving towards the electrolyte; (inset) photograph of a typical Sb_2_Se_3_ photocathode. *J*–*V* curves of the photocathodes under dark conditions and chopped sunlight illumination (c), and under dark conditions and continuous sunlight illumination (d). (e) The obtained HC-STH conversion efficiencies. (f and g) A comparison of our work with some state-of-the-art chalcogenide-based photocathodes. Statistical plots of M-20, M-30, and M-40 photocathodes, *i.e.*, (h) *J*_ph_, (i) *V*_on_, and (j) HC-STH conversion efficiencies.

Mo/Sb_2_Se_3_/CdS/MoS_2_ thin-film photocathodes with different MoS_2_ co-catalyst loading amounts (*i.e.*, 20 nm, 30 nm, and 40 nm) were prepared and studied; the corresponding devices were labelled as M-20, M-30, and M-40, respectively. Their PEC performance was measured using a classical 3-electrode PEC workstation, as shown in Fig. 1a. Upon light illumination, continuous visible hydrogen bubbles were observed on the photocathode surface, which migrated toward the acidic electrolyte (*i.e.*, 0.5 M H_2_SO_4_), indicating the efficient HER through water splitting (Fig. 1b). The as-prepared Sb_2_Se_3_ photocathodes of area 2 × 2 cm^2^ were quasi-homogenous, as shown in Fig. 1b (inset), suggesting great potential in scalable applications. Before performing PEC measurements, a defined active area of 0.95 cm^2^ was exposed using a water-resistant, light-resistant glue to ensure accurate and reproducible performance evaluation. Generally, the thickness of both the light-absorbing layer (Sb_2_Se_3_) and co-catalysts (MoS_2_) is very important for balancing charge carrier generation and HER activity. The current density–potential (*J*–*V*) curves of the M-20, M-30, and M-40 photocathodes under chopped light illumination and continuous light illumination are depicted in Fig. 1c and d, respectively. The M-20 photocathode with a 20 nm MoS_2_ layer exhibited relatively low PEC performance, with a low *J*_ph_ of ∼21.93 mA cm^−2^ at 0 *V*_RHE_. Moreover, its unsatisfactory HC-STH conversion efficiency of 2.22% was measured using the following equation:^2^

1 HC-STH (%) = *J*_ph_ × (*V*_RHE_ − *V*_H^+^/H_2__)/*P*_SUN_ × 100%

where *J*_ph_ is the photocurrent density at an applied potential of *V*_RHE_, *V*_H^+^/H_2__ represents the equilibrium redox potential for hydrogen (0 *V*_RHE_), and *P*_SUN_ denotes the sunlight intensity, set at 100 mW cm^−2^. This low PEC performance can be attributed to insufficient active sites, poor protection against photo-corrosion and higher recombination rates. When the MoS_2_ layer thickness was increased to 30 nm (M-30), an obvious increase in *J*_ph_ was observed, reaching ∼31.03 mA cm^−2^ (Fig. 1c), along with an impressive HC-STH conversion efficiency exceeding 3%. This optimized MoS_2_ could provide ideal surface coverage with more active sites and photoelectron transport pathways, while decreasing surface defects and recombination centers. It effectively achieved a balance between light absorption and catalytic activity, allowing efficient solar water splitting and hydrogen production. However, further increasing the MoS_2_ layer thickness to 40 nm (M-40) led to a decrease in *J*_ph_ (∼26.97 mA cm^−2^) and HC-STH efficiency (2.34%). This indicates that beyond a certain thickness, the MoS_2_ layer impeded light penetration into the Sb_2_Se_3_ absorber layer to generate charge carriers due to serious parasitic light absorption losses.^27^ To further validate the optimization, additional samples with 25 nm (M-25) and 35 nm (M-35) MoS_2_ thicknesses were investigated, both of which exhibited inferior PEC performance compared to M-30, with lower *J*_ph_ (∼25.84 mA cm^−2^ and 29.21 mA cm^−2^) and HC-STH efficiencies (2.60% and 2.75%) respectively (Fig. S2a and b†). These results further confirm that 30 nm represents the optimal MoS_2_ thickness for maximizing PEC performance in our Mo/Sb_2_Se_3_/CdS/MoS_2_ photocathode system. Additionally, another indicator of onset potential (*V*_on_), usually defined as the potential at which the steep *J*–*V* curves start, herein, was precisely measured by extrapolating the *J*–*V* curves in the quickly increasing regions (Fig. S3†). It is very important to mention that the obtained *J*_ph_ (∼31.03 mA cm^−2^) and *V*_on_ (∼0.43 *V*_RHE_) values belonging to the champion M-30 photocathode are obviously higher than those of previously reported MoS_2_-coated Sb_2_Se_3_-based photocathodes (Fig. 1f, g and Table 1). They also provide a comparative analysis of the PEC performance against previously reported state-of-the-art MoS_2_ co-catalyst-based Si, InP, GaP, and CZTS photocathodes; our device demonstrates comparable performance, especially with a higher *J*_ph_. Finally, the statistical distributions of different performance parameters are shown in Fig. 1h–j, presenting synchronous variation with key MoS_2_ thickness, confirming satisfactory reproducibility for efficient scalable solar hydrogen evolution applications.

**Table 1:** Summary of the PEC performances of different photocathodes using MoS_*x*_ compound as a co-catalyst

Photoelectrode	HER co-catalyst	Buffer solution	*J*_ph_ (mA cm^−2^)	*V*_on_ (*V*_RHE_)	HC-STH (%)	Stability (time, remain)	Reference
**Sb**_**2**_ **Se**_**3**_	**MoS**_**2**_	**pH 1**	**31.03**	**0.43**	**3.08**	**5 h, 90%**	**This work**
Sb_2_Se_3_	MoS_*x*_	pH 1	27	0.40	2.6	1.5 h, 93%	28
Sb_2_Se_3_	MoS_*x*_	pH 0	16	0.25	N/A	2 h, 70%	29
Sb_2_Se_3_	MoS_*x*_	pH 1	4.8	0.15	N/A	N/A	30
Sb_2_Se_3_	MoS_2_	pH 6.5	10	0.5	N/A	N/A	20
CZTS	MoS_*x*_	pH 3	18	0.60	3.0	10 h, 70%	27
Si	MoS_2_	pH 0	17.6	0.25	N/A	3 h, 78%	31
GaP	MoS_*x*_	pH 0	6.4	0.71	1.50	N/A	32
p-Si	MoS_2_	pH 1	42.3	0.72	0.64	15 h	33
n^+^n p^+^-Si	MoS_2_	pH 1	36.34	0.5	5.5	10 h	34
InP	MoS_3_	pH 0	22	0.6	6.4	1 h, 90%	35

**Fig. 2:**
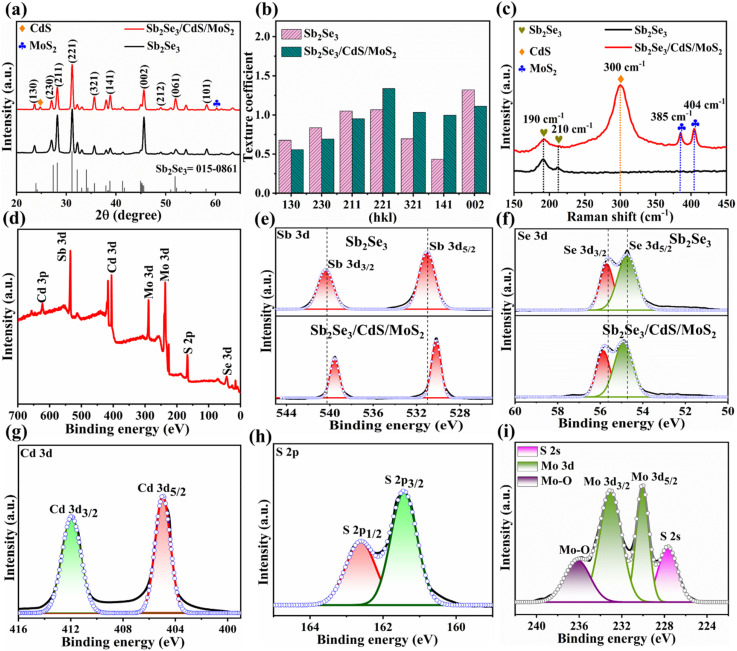
(a) XRD, (b) texture coefficient of XRD peaks, and (c) Raman spectra of bare Sb_2_Se_3_ and Sb_2_Se_3_/CdS/MoS_2_ samples. (d) XPS survey graph of Sb_2_Se_3_/CdS/MoS_2_, the binding energy displacement of (e) Sb and (f) Se. XPS spectra for (g) Cd 3d, (h) S 2p, and (i) Mo 3d.

A detailed morphological and structural investigation was first carried out to validate the quality of the Sb_2_Se_3_ light-absorbing thin film, the interface modification *via* MoS_2_ as a co-catalyst, and its impact on PEC performance of the device. The XRD patterns of the pure Sb_2_Se_3_ and Sb_2_Se_3_/CdS/MoS_2_ thin films are presented in Fig. 2a. The high purity and crystallinity of the as-deposited Sb_2_Se_3_ films are confirmed by the presence of four major diffraction peaks, corresponding to the (211), (221), (321), and (002) planes, consistent with the standard Sb_2_Se_3_ (JCPDS Card No. 15-0861). These distinct peaks suggest the formation of a highly crystalline light-absorbing layer, which is crucial for minimizing defect states and optimizing charge carrier mobility within the film. After deposition of CdS and MoS_2_, two additional peaks appear at 24.97° and 58.9° corresponding to the (111) plane of CdS and (110) plane of MoS_2_, according to JCPDS Card No. 41-1049 and JCPDS Card No. 37-1492, respectively. The peak observed for MoS_2_ is assigned to pure MoS_2_ highlighted in Fig. S4a,† while its compact granular structure can also be observed from the SEM image of MoS_2_ in Fig. S4d†. The (111) plane of CdS, known for its optimal electron transport properties, combined with the catalytic (110) plane of MoS_2_, suggests, improved charge transfer and catalytic activity at the semiconductor/electrolyte interface, likely contributing to enhanced PEC performance observed in the Sb_2_Se_3_/CdS/MoS_2_ photocathode.^36^ The obtained results were further confirmed by analyzing the texture coefficient (TC) to examine the preferred orientation in Sb_2_Se_3_ and Sb_2_Se_3_/CdS/MoS_2_ thin films, using the following equation:^37^2
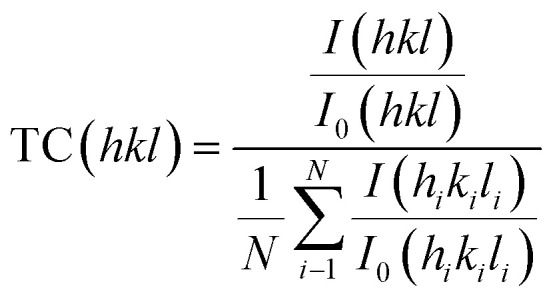
where *N*, *I*(*hkl*), and *I*_0_(*hkl*) show the peak number utilised during calculations, the intensity of the measured diffraction, and the reported diffraction intensity (PDF card), respectively. Fig. 2b illustrates an overall increase in the TC of (*hk*1) planes, coupled with a decrease in the TC of the (*hk*0) planes after the deposition of CdS and MoS_2_ layers on the Sb_2_Se_3_ thin film, suggesting a reorientation of the crystallographic structure of Sb_2_Se_3_ driven by the post-deposition processes. The improvement of the (*hk*1) planes is typically associated with optimal carrier transport and high charge mobility, resulting improved charge transfer pathways in the Sb_2_Se_3_/CdS/MoS_2_ photocathode.^37^Fig. 2c presents the Raman spectra of pure Sb_2_Se_3_ and Sb_2_Se_3_/CdS/MoS_2_ thin-film photocathodes. In the Sb_2_Se_3_ spectrum, the most prominent peak of Sb_2_Se_3_ phase around 190 cm^−1^ is observed, which refers to the A_2u_ mode of the Sb–Sb bond, one of the most common vibrational modes in the Sb_2_Se_3_ phase. The additional peak around 210 cm^−1^ corresponds to Sb–Se bond vibration within the Sb_2_Se_3_ unit. In the Sb_2_Se_3_/CdS/MoS_2_ spectrum, an intense peak appears around 300 cm^−1^ corresponding to the longitudinal optical mode of the hexagonal CdS buffer layer. Moreover, two additional peaks at about 385 cm^−1^ and 405 cm^−1^ are observed, which correspond to the in-plane E_2g_ and out-plan A_1g_ modes of MoS_2_, respectively.^38^ The presence of these characteristic peaks in Raman spectra of the pure as-deposited MoS_2_ thin film (Fig. S4c†) confirms the successful deposition and integration of the crystalline MoS_2_ layer within the Sb_2_Se_3_/CdS/MoS_2_ photocathode via the PVD process. Importantly, its layered structure and beneficial crystallographic orientation might interact synergistically with the Sb_2_Se_3_/CdS layers to reduce the photo-corrosion effect, and provide a substantial decrease in charge transfer resistance.^39^

X-ray photoelectron spectroscopy (XPS) investigations were performed to better understand the chemical composition. Fig. 2d shows the XPS survey spectra of Sb_2_Se_3_/CdS/MoS_2_. Fig. 2e demonstrates the Sb 3d spectra with binding energies b/w 525 and 545 eV for both bare Sb_2_Se_3_ and Sb_2_Se_3_/CdS/MoS_2_. The separation between the peaks of Sb 3d doublet pairs was retained at 9.58 eV.^40^ It was also confirmed from the Se spectra shown in Fig. 2f, that two doublets of Se (3d_5/2_ and 3d_3/2_), separated by 0.78 eV energy, showing the presence of Se^2−^. Moreover, Fig. 2e reveals a prominent shift in the Sb 3d peak toward lower binding energy in the case of Sb_2_Se_3_/CdS/MoS_2_. As an explanation, at the Sb_2_Se_3_/CdS/MoS_2_ interface, the higher Fermi levels of n-type CdS and MoS_2_ cause electron flow to p-type Sb_2_Se_3_ until equilibrium is reached, resulting in band bending, with Sb_2_Se_3_ bands shifting upward and the CdS/MoS_2_ bands bending downward, also confirming the favorable formation of the Sb_2_Se_3_/CdS/MoS_2_ junction. Two notable peaks positioned at 412.2 eV and 405 eV (shown in Fig. 2g) are assigned to Cd 3d_3/2_ and 3d_5/2_ respectively. Fig. 2h shows two distinguished peaks located at 162.8 eV and 161.5 eV, which correspond to S 2p_1/2_ and S 2P_3/2_ respectively. The Mo 3d and S 2s core level regions, with binding energies ranging from 222 to 242 eV, are depicted in Fig. 2i. The Mo 3d doublet peaks demonstrating the presence of Mo^4+^ (Mo 3d_5/2_ and Mo 3d_3/2_) appear around 229 and 232 eV, respectively. These peaks are characteristic of the MoS_2_ structure, confirming that Mo occurs primarily in its active Mo^4+^ state, which is important for promoting efficient charge transfer and catalyzing the HER. Furthermore, the S 2s peak around 226 eV confirms the presence of sulfur (S^2−^) in the MoS_2_ layer.^41^ The Mo 3d third peak around 235 eV corresponding to the Mo^6+^ state of MoO_3_ shows the presence of a Mo oxide state in MoS_2_. The presence of MoO_3_ shows the oxidation of the MoS_2_ layer, which may results from sulfur vacancies during deposition or exposure to air, where sulfur vacancies react with oxygen, or from interactions between Mo and the substrate surface.^42^

**Fig. 3:**
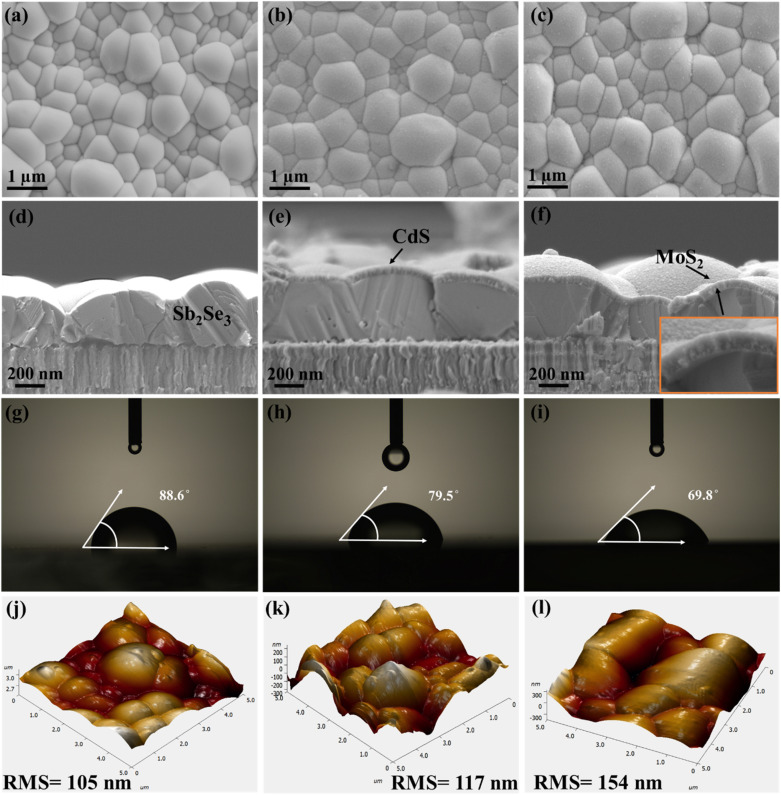
SEM micrographs of (a) Sb_2_Se_3_, (b) Sb_2_Se_3_/CdS, and (c) Sb_2_Se_3_/CdS/MoS_2_ samples. (d–f) The corresponding cross-sectional SEM images. (g–i) The measured CAs (using H_2_SO_4_ electrolyte droplets) of the samples, and (j–l) AFM images of the sample surfaces.

Scanning electron microscopy (SEM) images show the surface morphologies of Sb_2_Se_3_, Sb_2_Se_3_/CdS and Sb_2_Se_3_/CdS/MoS_2_ thin films depicted in Fig. 3a–c. Additionally, the corresponding energy dispersive spectroscopy (EDS) information is presented in Fig. S5a–c,† which supports the elemental composition. The smooth surface morphology of pristine Sb_2_Se_3_ becomes rougher gradually after the deposition of the CdS and MoS_2_ layers. This enhanced surface roughness indicates the successful deposition of CdS and MoS_2_ layers and is predicted to have an impact on the photocathode's light absorption and charge carrier dynamics. The cross-sectional SEM images (Fig. 3d–f) confirm the compact deposition of all three layers. MoS_2_ forms a conformal layer over the CdS-coated Sb_2_Se_3_ surface, potentially covering grain boundaries and interfaces without significantly altering the surface topography. This homogeneous distribution of MoS_2_ is very important for enhancing charge separation and creating efficient electron transport pathways, thus enhancing PEC performance.^27^ Wettability is an important parameter assessed by measuring the contact angle (CA) between solid and fluid surfaces and can be classified as completely wettable (CA = 0°), hydrophobic or poorly wettable (CA > 90°) and hydrophilic or partially wettable (CA < 90°).^40^ The wettability of Sb_2_Se_3_, Sb_2_Se_3_/CdS, and Sb_2_Se_3_/CdS/MoS_2_ (M-30) samples was examined through CAs formed between the sample surfaces and H_2_SO_4_ electrolyte droplets. According to Fig. 3g–i, the Sb_2_Se_3_, Sb_2_Se_3_/CdS, and Sb_2_Se_3_/CdS/MoS_2_ (M-30) sample surfaces exhibit hydrophilic nature with CAs of 88.6°, 79.5°, and 69.8°, respectively. To further validate this, the CAs of 89.2°, 67.6°, and 56.3° for the same samples were also measured by using glycerol droplets which display similar trends as depicted in Fig. S7a–c.† Importantly, the M-30 sample depicts the minimum CA, demonstrating a significant enhancement in wettability after MoS_2_ deposition. Additionally, the M-20 and M-40 samples display higher CAs values of 76.9° and 72.2° (Fig. S6a and b†), respectively, which are still lower than that of Sb_2_Se_3_/CdS but higher than that of the M-30 sample which suggests that the M-30 sample achieves optimal wettability among the other samples. This variation is closely related to changes in roughness, surface tension of liquids, free energy, and grain size of the solid surface. Such enhanced wettability improves the PEC performance of photocathodes by increasing the number of exposed reaction sites and enabling photoelectron transfer.^26^ The roughness and surface morphologies of the corresponding Sb_2_Se_3_, Sb_2_Se_3_/CdS, and Sb_2_Se_3_/CdS/MoS_2_ thin films were studied using atomic force microscopy (AFM) (Fig. 3j–l). Additionally, 2D and 3D images of the pure MoS_2_ thin film are shown in Fig. S9a and b.† The average root mean square (RMS) surface roughness value of Sb_2_Se_3_ is 105 nm, followed by 117 nm for Sb_2_Se_3_/CdS, and 154 nm for the Sb_2_Se_3_/CdS/MoS_2_ (M-30) thin film surface. Notably, the M-20 and M-40 samples exhibit RMS roughness values of 129 nm and 146 nm, respectively (Fig. S8a and b†), which are lower than that of the M-30 sample. This indicates that the M-30 sample has the most textured surface among the MoS_2_-coated samples, which may contribute to its higher PEC performance. Thus, it can be speculated that the improvement in wettability of Sb_2_Se_3_/CdS/MoS_2_ might be result from the evolution of surface roughness, which matches well with the roughness-dependent wettability model presented by Wenzel, *i.e.*, the roughness will make a naturally hydrophilic surface more hydrophilic.^43^

**Fig. 4:**
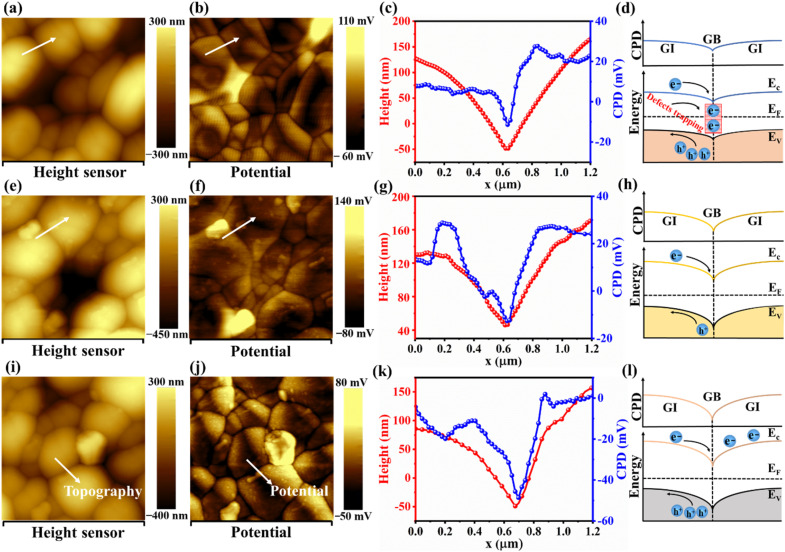
KPFM scanning surface topography and CPD maps for (a and b) Sb_2_Se_3_, (e and f) Sb_2_Se_3_/CdS, and (i and j) Sb_2_Se_3_/CdS/MoS_2_ samples, respectively. (c, g and k) The corresponding topography and potential line scans acquired from the white arrow lines. (d, h and l) Schematic of the energy band structure and CPD near the GBs, where “e^−^” represents electrons; “h^+^” represents holes.

Kelvin probe force microscopy (KPFM) analysis was then performed; this advanced technique allowed us to study the topography and contact potential difference (*V*_CPD_) across thin films, providing critical insights into their surface properties. Fig. 4a, e and i show the micrographs, and Fig. 4b, f, and j show the surface potential distributions for Sb_2_Se_3_, Sb_2_Se_3_/CdS, and Sb_2_Se_3_/CdS/MoS_2_ samples, respectively. The AFM images indicate that the surface morphology of the Sb_2_Se_3_ thin films remains consistent across different layer depositions, which correlates well with the top-view SEM micrographs (Fig. 3a–c). The pronounced contrast in CPD between grain interiors and boundaries indicates a strong intergranular carrier extraction, which facilitates efficient charge separation and carrier transport, contributing to enhanced PEC performance. The white arrow line scans, which show CPD variations for Sb_2_Se_3_, Sb_2_Se_3_/CdS, and Sb_2_Se_3_/CdS/MoS_2_ (M-30), are depicted in Fig. 4c, g, and k, and those for M-20 and M-40 are shown in Fig. S10a and b,† respectively; these scans show the electronic landscape of the samples. The CPD values for Sb_2_Se_3_, Sb_2_Se_3_/CdS, and Sb_2_Se_3_/CdS/MoS_2_ samples lie in the range of −50 to 30 mV, and a similar decrease in CPD from the grain interior (GI) to the grain boundary (GB) can be observed. This indicates that the sample work function at the GB is lower, *i.e.*, the Fermi energy level *E*_F_ at the GB is closer to the vacuum level, leading to a downward band bending at the GB. The corresponding schematic diagrams of the energy band structure are presented in Fig. 4d, h and l. Upon further comparison, the average potential difference between the GB and GI for Sb_2_Se_3_, Sb_2_Se_3_/CdS, and Sb_2_Se_3_/CdS/MoS_2_ are 23, 35, and 45 mV, respectively. Thus, the increased band bending after the deposition of CdS and MoS_2_ would effectively separate electron–hole pairs by attracting electrons (minority carriers) towards the GBs while promoting holes (majority carriers) to flow into GIs. This mechanism is very important for improving electron extraction and minimizing electron–hole recombination, as well as guiding the charge carriers towards the interface to improve photocurrent generation. Thus, it is predicted to contribute to a higher *J*_ph_ in photocathode, improving the PEC performance.^44^

**Fig. 5:**
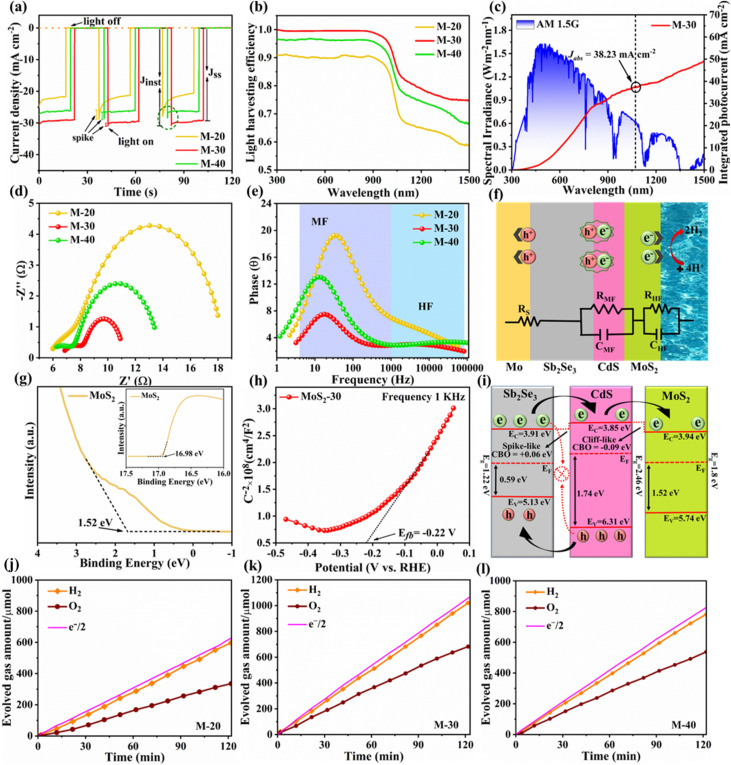
(a) Transient photocurrent response of M-20, M-30, and M-40 photocathodes. (b) Light harvesting efficiency (LHE) of M-20, M-30 and M-40 photocathodes. (c) Energy density flux for the AM 1.5G standard solar spectrum and integrated *J*_ph_ of the M-30 sample. (d) Nyquist plots and (e) the equivalent Bode plots of M-20, M-30, and M-40 photocathodes. (f) Schematic diagram along with the corresponding equivalent circuit system related to charge carrier dynamics procedures. (g) UPS characterization derived *V*_B_ positions and SEC edges of the MoS_2_ thin film. (h) M–S plots of the pure MoS_2_-30 thin film at a frequency of 1 kHz. (i) Schematic energy band alignment of the Sb_2_Se_3_/CdS/MoS_2_ photocathode. H_2_ generation amount as a function of AM 1.5G solar light illumination time over the (j) M-20, (k) M-30, and (l) M-40 photocathodes at 0 *V*_RHE_ for 2 h in pH 1 buffer solution.

The photocurrent response and charge transport kinetics of the Sb_2_Se_3_-based photocathodes were thoroughly examined. In Fig. 5a, the transient photocurrent decay spectra show that M-20 and M-40 photocathodes exhibited a prominent “spike-like” transient behavior upon light illumination, indicating substantial charge carrier recombination since the temporarily accumulated photoelectrons might easily recombine before reaching a steady state. Conversely, the M-30 photocathode exhibits a very small “spike-like” transient, suggesting reduced charge recombination and improved carrier transport, which is closely related to its optimized surface co-catalyst coverage and photoelectron transport.^26^ Specifically, the charge transfer efficiency (*η*_tran_) is defined as the ratio of the steady-state photocurrent density (*J*_ss_) to the instantaneous photocurrent density (*J*_inst_). The charge separation efficiency (*η*_sep_) can also be obtained using the following equations:^26,45^3
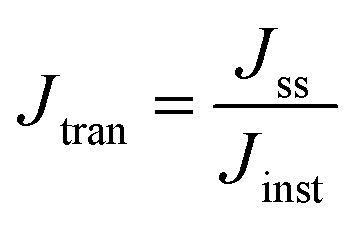

4 *J*_ph_ = *J*_abs_ × *η*_tran_ × *η*_sep_

As a result, the M-30 photocathode achieved the highest *η*_tran_ of 97.31%, as compared to 81.39% and 93.05% for M-20 and M-40, respectively. In parallel, the *η*_sep_ for M-30 was also the highest at 82.04%, while M-20 and M-40 exhibited lower values of 51.42% and 69.74%, respectively. The optimized M-30 device simultaneously achieved high charge separation and transport, resulting in improved PEC performance. The theoretical *J*_ph_ (*i.e.*, *J*_abs_) of the Sb_2_Se_3_-based photocathodes was also calculated (Fig. 5b, c, S11a, b and Note S1, ESI†) by using the standard solar spectrum (AM 1.5G) and wavelength-dependent light harvesting efficiency (LHE), and assuming 100% conversion of the absorbed photons to current density. Since *λ*_e_ is ≈1070 nm, the calculations show that the M-30 photocathode achieves a theoretical *J*_abs_ of 38.23 mA cm^−2^ (Fig. 5c), which is the highest among the three samples (*J*_abs_ values of 30.04 mA cm^−2^ and 33.82 mA cm^−2^ for M-20 and M-40, respectively). The enhanced *J*_abs_ for M-30 can be attributed to its ideal MoS_2_ thickness and light absorption capacity. Photoelectrochemical impedance spectroscopy (PEIS) investigation of Sb_2_Se_3_-based photocathodes was conducted to evaluate the charge transfer resistance and specific charge carrier recombination mechanisms within the system. Fig. 5d represents the Nyquist plots for M-20, M-30, and M-40 photocathodes, displaying two distinct arcs for each sample, consistent with the corresponding Bode plots shown in Fig. 5e. The Bode diagram illustrates two different regions, covering the low and high frequencies in the range of 1 to 100 kHz. The PEIS data were fitted using an equivalent circuit model consisting of a series resistance (*R*_S_) and two pairs of parallel resistor–capacitor (*R*–*C*) elements, as shown in Fig. 5f. The as-fitted results for M-20, M-30, and M-40 photocathodes are presented in Table S1.† In Fig. 5f, *R*_S_ represents the series resistance at the Mo/Sb_2_Se_3_ back contact interface, and the comparable *R*_S_ values (≈5–6 Ω) across the device indicated a well-established and favorable back interface contact. The high-frequency arc-derived *R*_HF_ and *C*_HF_ reflect the charge transfer resistance at the semiconductor heterojunction interface and the associated capacitance within the space charge region. In contrast, *R*_LF_ and *C*_LF_ (low frequency arc derivatives) represent the electrochemical charge transfer/reaction resistance within the Helmholtz layer at the electrode–electrolyte interface, along with the corresponding interface/surface-states capacitance.^46^ Notably, the smaller *C*_HF_ value (5.03 × 10^−6^ F) for the M-30 photocathode implies a shorter charge accumulation time, while the reduced *R*_HF_ value suggests less interface defects and more effective charge carrier transport and separation efficiencies at the Sb_2_Se_3_/CdS heterojunction interface. Furthermore, the minimum *R*_LF_ value of 4.599 Ω for M-30 as compared to 16.955 Ω for M-20 and 7.929 Ω for M-40 counterparts indicates more efficient HER at the electrode–electrolyte interface under suitable MoS_2_ co-catalyst loading.

Bulk and interface charge carrier dynamic characteristics strongly affect the PEC performance of the Sb_2_Se_3_-based thin films photocathodes. Therefore, ultraviolet photoelectron spectroscopy (UPS) characterization was performed to examine the energy band alignment of the Sb_2_Se_3_ absorber, CdS buffer layer (Fig. S11c and d†) and MoS_2_ (Fig. 5g) thin films to further understand the recombination, transport and separation of charge carriers. According to the secondary electron cut-off (SEC) edge and valence band (*V*_B_) position, the conduction band (*E*_C_) of MoS_2_ is −3.94 eV. Compared with the normal hydrogen electrode (NHE), it is −0.50 *V*_NHE_, with the conversion implemented by using the following formula:^47^

5 Energy = −*eE*_appl_(*vs.* NHE) − 4.44 eV

The conduction band minimum (CBM) of the MoS_2_ thin film is located above the hydrogen reduction potential, which thermodynamically enhances water reduction with hydrogen evolution through solar water splitting, resulting in an enhanced *J*_ph_. Mott–Schottky (M–S) measurements were further performed to investigate the capacitance of MoS_2_ thin films by examining film capacitance (*C*) at the semiconductor/electrolyte interface as a function of the applied potential (*V*). Fig. 5h, S12a and b† demonstrate that 1/*C*^2^ increases with potential *V* in the presence of the space charge region (SCR), demonstrating evident n-type characteristics for MoS_2_ films.^48^ Furthermore, the acceptor density (*N*_A_) and flat band potential (*E*_fb_) were calculated using the following equation:^2^6
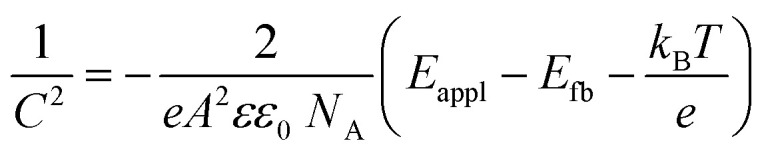
where *e* is the electron unit, *ε* is the relative dielectric constant, *ε*_0_ is the vacuum permittivity, *T* represents the temperature, *k* represents the Boltzmann constant, *A* represents the active device area, *N*_A_ represents the accepter density, and *C* is the SCR capacitance forms at the semiconductor–electrolyte interface. The analysis shows that the pure MoS_2_-30 (30 nm) thin film exhibited the lowest *N*_A_ (3.54 × 10^17^ cm^−3^), compared to pure MoS_2_-20 (20 nm) (4.21 × 10^18^ cm^−3^) and pure MoS_2_-40 (40 nm) (7.81 × 10^17^ cm^−3^) (Fig. S12a and b†). This lower *N*_A_ value in pure MoS_2_-30 suggests fewer traps for charge carriers, enhanced charge dynamics and a larger space charge region, which together improve charge separation efficiency and reduce recombination losses at the optimal thickness. The valence band (*V*_B_) position of MoS_2_ can also be calculated from *N*_A_, *E*_fb_, effective mass of hole 
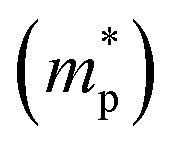
, and valence band effective density of states function (*N*_v_), by using the following formula:^49^7
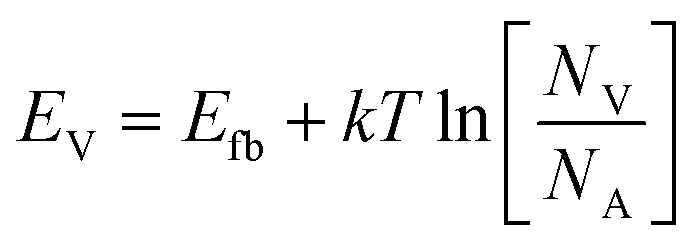
8
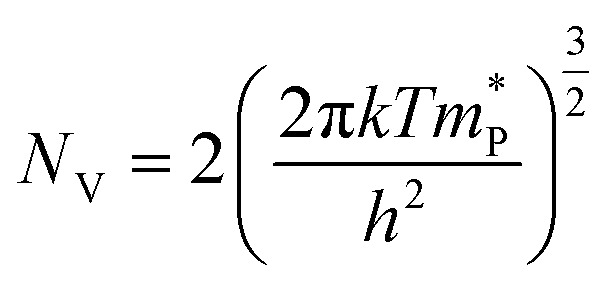


The MoS_2_ thin film possesses a similar *E*_V_ value (−5.74 eV), which matches well with the UPS-derived *E*_V_ (−5.74 eV). Finally, band bending and energy band alignment for Sb_2_Se_3_/CdS/MoS_2_ are depicted in Fig. 5i. The CdS and MoS_2_ thin films exhibit stronger n-type conductive characteristics, suggesting that MoS_2_ would increase the electron carrier density. The valence band offset (VBO) values for n-CdS/p-Sb_2_Se_3_ and n-CdS/n-MoS_2_ are 1.18 eV and 0.57 eV, respectively, which are similar to those reported in previous work (1.14 ± 0.10 eV);^50^ these values provide the required energy barrier that efficiently stops photo-generated holes from moving toward the electrode–electrolyte interface for recombination. The conduction band alignment between Sb_2_Se_3_ and CdS exhibits a “spike-like” band alignment, with a positive conduction band offset (CBO of +0.06 eV), which falls within the optimal range of 0–0.4 eV, suitable for high quality semiconductor heterojunctions. This band alignment decreases energy barriers, enhances charge carrier separation and transport while reducing recombination. After that, the electrons migrate from CdS to the MoS_2_ layer, which has a slightly higher conduction band edge, further facilitating electron flow. MoS_2_ acts as a co-catalyst and provides active sites for the HER at the photocathode surface. This layered band structure, with suitable band-bending, confirms efficient electron transport from Sb_2_Se_3_ through CdS to MoS_2_, while holes are directed toward the back contact. Thus, the MoS_2_ layer improves charge separation and enables effective utilization of photo-generated carriers for hydrogen production, increasing overall PEC efficiency.

Gas chromatography (GC) was used to *in situ* measure the production of H_2_ and O_2_, and then evaluate the photon utilization capability and faradaic efficiency. As shown in Fig. 5j–l, the champion M-30 photocathode demonstrated continuous H_2_ and O_2_ evolution at rates of 8.50 and 5.68 μmol min^−1^, respectively, which are higher than those of M-20 (4.97 and 2.79 μmol min^−1^) and M-40 (6.51 and 4.47 μmol min^−1^) photocathodes. Faradaic efficiencies of 91%, 94% and 92% (calculated from the ratio of H_2_ to e^−^/2) were obtained for M-20, M-30, and M-40 photocathodes, indicating minimal side reactions or competing redox processes on the photoelectrode surface. After 2 h of continuous irradiation, approximately 1020 μmol of H_2_ and 682 μmol of O_2_ were generated by the champion M-30 photocathode, further confirming the durability and versatility of the champion M-30 device for PEC processed solar water splitting. The recorded video in 0.5 M H_2_SO_4_ buffer solution under AM 1.5G continuous sunlight irradiation during operation shows continues hydrogen production (Movie S1, ESI†), confirming the excellent PEC performance of the device.

**Fig. 6:**
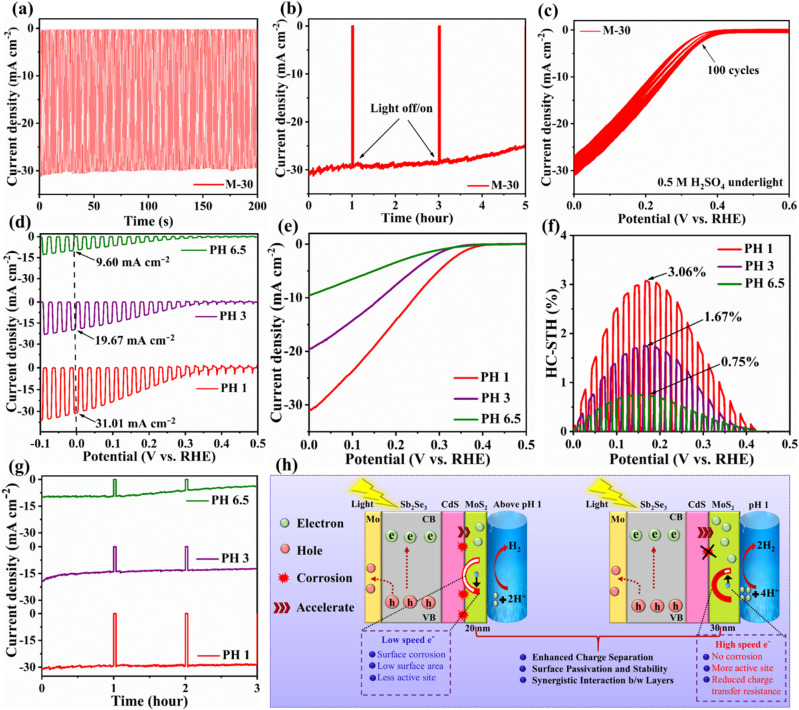
(a) *J*–*T* curve of the M-30 photocathode at 0 *V*_RHE_ under AM 1.5G simulated solar irradiation. (b) Photocurrent stability experiment of the champion M-30 photocathode at 0 *V*_RHE_ under continuous sunlight illumination within 5 hours. (c) CV measurement of the M-30 photocathode in 0.5 M H_2_SO_4_ under illumination. *J*–*V* curves of the M-30 photocathode at different pH solution, and (d) under chopped light, and (e) under continuous light irradiation. (f) The calculated HC-STH conversion efficiencies. (g) Stability of M-30 photocathodes in pH 1, pH 3, and pH 6.5 solution under AM 1.5G simulated light irradiation. (h) Mechanism schematic representation of the PEC performance Mo/Sb_2_Se_3_/CdS/MoS_2_ photocathodes.

After the successful demonstration of MoS_2_ as an efficient HER co-catalyst, its secondary role as a protective layer was also examined. The current density–time (*J*–*T*) curves for those three devices are shown in Fig. 6a, S13a and b,† under AM 1.5G simulated sunlight illumination at 0 *V*_RHE_. As illustrated in Fig. 6a, when the light was switched on, the *J*_ph_ of M-30 quickly increased from 0 mA cm^−2^ to 31.03 mA cm^−2^, and when the light was turned off, it quickly returned to its initial value. This fast and stable response demonstrates the excellent reversibility and stability of the MoS_2_-based Sb_2_Se_3_ photocathodes. In addition to developing a highly efficient photocathode, assessing its real long-term PEC stability is also crucial for practical applications. Fig. 6b displays the photocurrent stability of the champion device (M-30) over 5 h at 0 *V*_RHE_ under AM 1.5G continuous light irradiation in an H_2_SO_4_ buffer solution (pH 1), while Fig. S14a and b† show the stability tests for M-20 and M-40 devices. The thinner MoS_2_ layer in M-20 likely provides insufficient surface protection due to limited surface coverage to directly expose part of the underlying Sb_2_Se_3_/CdS layer to the electrolyte, leading to a higher rate of photo-corrosion and reduced PEC stability. By contrast, the champion M-30 photocathode demonstrated remarkable stability over the 5 h testing period, retaining approximately 90% of its initial *J*_ph_ under continuous light irradiation. The LSV curves and PEC performance before and after the stability test are shown in Fig. S14c, d and Movie S2.† Its excellent stability is closely related to the MoS_2_ layer with appropriate thickness, which effectively protects the Sb_2_Se_3_/CdS surface, avoiding localized dissolution that could induce thermodynamic photo-corrosion and degradation during prolonged operation. This finding is consistent with other studies in which Sb_2_Se_3_ photocathodes were protected by additional layers like C_60_ or TiO_2_ to prevent CdS degradation.^26^ Table S2† presents a comparative analysis of the PEC performance and stability of Sb_2_Se_3_-based photocathodes using MoS_2_ and Pt as co-catalysts. This comparison highlights the effectiveness of MoS_2_ as a low-cost alternative to noble metal catalysts while maintaining competitive PEC activity and durability. To further evaluate the intrinsic stability and lifespan of the Sb_2_Se_3_ absorber (Fig. S13c†), bare MoS_2_ film (Fig. S13d†), and M-30 thin-film photocathode (Fig. 6c), a photo-corrosion stress test was conducted using cyclic voltammetry. No significant photo-corrosion currents or redox peaks were observed in any of the samples after 100 cycles, strongly suggesting their exceptional tolerance to photo-corrosion during practical PEC applications. As shown in Fig. 6d–g, the PEC performance and stability of the champion M-30 photocathode also varies significantly across different pH buffer solutions. Specifically, it exhibited a *J*_ph_ of 31.01, 19.67, and 9.60 mA cm^−2^, and HC-STH efficiencies of 3.06%, 1.67%, and 0.75% in pH 1, pH 3, and pH 6.5 buffer solutions, respectively, confirming the faster and more efficient HER in acidic buffer solution. Moreover, its photocurrent stability (3 h duration) under acidic conditions (pH 1) is superior to that in neutral (pH 6.5) electrolytes. It is similar to some previous reports, *e.g.*, MoS_*x*_ co-catalyst modified CZTS and InP photocathodes, both of which exhibit high photocurrent and good stability in acidic conditions as compared to neutral buffer solution.^29,35^ As shown in Fig. 6h, this behavior can be described by the fact that, in acidic environments with a higher concentration of H^+^ ions, photo-excited electrons can more easily and efficiently combine ionically to produce hydrogen gas.^51^ The rapid transport of photo-excited electrons in an acidic buffer solution could also mitigate the photo-corrosion of the photoelectrode, and hence increase the PEC stability of the photocathode.^3^ These findings are particularly important for optimizing photocathode design for practical solar-driven hydrogen production applications. The enhanced PEC performance under acidic conditions expands the potential applications of these photocathodes to electrocatalysis, sensors, and environmental remediation, where stability under harsh conditions is often a critical requirement.

## 4. Conclusion

In this work, we successfully demonstrated the enhancement of Sb_2_Se_3_-based photocathodes for solar hydrogen generation by the introduction of MoS_2_ as a cost-effective, noble-metal-free co-catalyst. The optimized Sb_2_Se_3_/CdS/MoS_2_ photocathode achieved a record *J*_ph_ of 31.03 mA cm^−2^ at 0 *V*_RHE_ and a HC-STH efficiency of 3.08% in a pH 1 buffer solution. The MoS_2_ co-catalyst played an important role in increasing the overall performance by decreasing photo-corrosion, electron–hole recombination, and charge transfer resistance at the semiconductor/electrolyte interface. Our systematic investigation shows that MoS_2_ not only facilitated efficient charge carrier transport but also improved light-harvesting efficiency by optimizing the band alignment at the Sb_2_Se_3_/CdS interface. This dual function of MoS_2_, as a co-catalyst and a protective layer, contributed to a stable, high-performance photocathode capable of sustained hydrogen evolution over extended periods under acidic conditions. Additionally, the photocathode exhibited improved stability and performance across a range of pH conditions, making it a promising candidate for practical applications in solar hydrogen production. In short, the combination of Sb_2_Se_3_ and MoS_2_ offers a feasible, eco-friendly alternative to Pt-based devices, advancing the development of efficient and cost-effective photocathodes for hydrogen production.

## Data availability

The data supporting this article have been included as part of the ESI.†

## Author contributions

Munir Ahmad: data curation, investigation, methodology, conceptualization, writing – original draft. Anadil Gul: methodology, investigation. Hafiz Sartaj Aziz: methodology, investigation. Tahir Imran: data curation, formal analysis. Muhammad Ishaq: data curation, investigation. Muhammad Abbas: data curation, investigation. Zhenghua Su: methodology, investigation. Shuo Chen: conceptualization, formal analysis, writing – review & editing, supervision, funding acquisition.

## Conflicts of interest

The authors declare no conflict of interest.

## Supplementary Material

SC-OLF-D5SC01663K-s001

SC-OLF-D5SC01663K-s002

SC-OLF-D5SC01663K-s003
