# Supplementary material for: Pt-free MoS2 co-catalyst enables record photocurrent density in Sb2Se3 photocathodes for highly efficient solar hydrogen production
Source: Chem Sci. 2025 Apr 16;16(20):8946–58. doi: 10.1039/d5sc01663k (PMC12013506; doi:10.1039/d5sc01663k)
Supplement: SC-016-D5SC01663K-s003 [file SC-016-D5SC01663K-s003.pdf]

Supplementary Information (SI) for Chemical Science.
This journal is © The Royal Society of Chemistry 2025

Supporting Information

Pt-Free MoS_2 Co-Catalyst Enables Record Photocurrent Density in Sb_2Se_3 Photocathodes for Highly Efficient Solar Hydrogen Production

Munir Ahmad[a], Anadil Gul[b], Hafiz Sartaj Aziz[a], Tahir Imran[a], Muhammad Ishaq[a], Muhammad Abbas[a], Zhenghua Su[a], and Shuo Chen [a,*]

[a]Institute of Thin Film Physics and Applications, Shenzhen Key Laboratory of Advanced Thin Films and Applications, Key Laboratory of Optoelectronic Devices and Systems of Ministry of Education and Guangdong Province, State Key Laboratory of Radio Frequency Heterogeneous Integration, College of Physics and Optoelectronic Engineering, Shenzhen University Shenzhen 518060, China

[b]College of Health Science and Environmental Engineering, Shenzhen Technology University Shenzhen 518118, China

*Corresponding Author– Shuo Chen; chensh@szu.edu.cn

Table S1. Summary of PEIS fitted parameters for M-20, M-30, and M-40 photocathodes.

Device	R_S (Ω)	R_{HF} (Ω)	C_{HF} (F)	R_{MF} (Ω)	C_{MF} (F)
M-20	5.635	2.939	3.815×10^{-4}	16.955	1.476×10^{-3}
M-30	5.093	1.372	5.03×10^{-6}	4.599	1.627×10^{-3}
M-40	5.342	2.572	5.254×10^{-5}	7.929	1.221×10^{-3}

Table S2. Comparison of the PEC performances and stability of photocathodes using MoS_2 and Pt as a co-catalyst.

Photoelectrode	Electrolyte	J_{ph} (mA cm^{-2})	V_{on} (V_{RHE})	HC-STH (%)	Stability (time, remain)	Refs
**Mo/Sb_2Se_3/CdS/ MoS_2**	**0.5M H_2SO_4**	**31.03**	**0.43**	**3.08**	**5 h, 90%**	**This work**
Mo/Sb_2Se_3/TiO_2/Pt	1M H_2SO_4	20.2	0.57	1.36	2 h, 85%	[1]
Mo/grad/Sb_2Se_3/TiO_2/Pt	1M H_2SO_4	14.2	0.42	2	2 h, 70%	[2]
FTO/Au/Sb_2Se_3/TiO_2/Pt	0.5M H_2SO_4	11.3	0.3	0.53	2 h, 50%	[3]
FTO/Au/Sb_2Se_3/PABA/TiO_2/Pt	1M H_2SO_4	35	0.50	4.79	5h, 15%	[4]
FTO/Au/Sb_2Se_3/CdS/TiO_2/Pt	0.1M H_2SO_4	19	0.50	3.4	5 h, 35%	[5]
FTO/Au/Sb_2Se_3/CdS/TiO_2/Pt	0.5M H_2SO_4	11	0.47	N/A	3 h, 73%	[6]
Mo/Sb_2Se_3/CdS/Pt	0.5M H_2SO_4	16.25	0.52	2.58	1 h, 87%	[7]
Mo/Sb_2Se_3/$Cd_{0.5}Zn_{0.5}S$/Pt	0.2M Na_2HPO_4	17.5	0.80	2.19	2 h, 80%	[8]

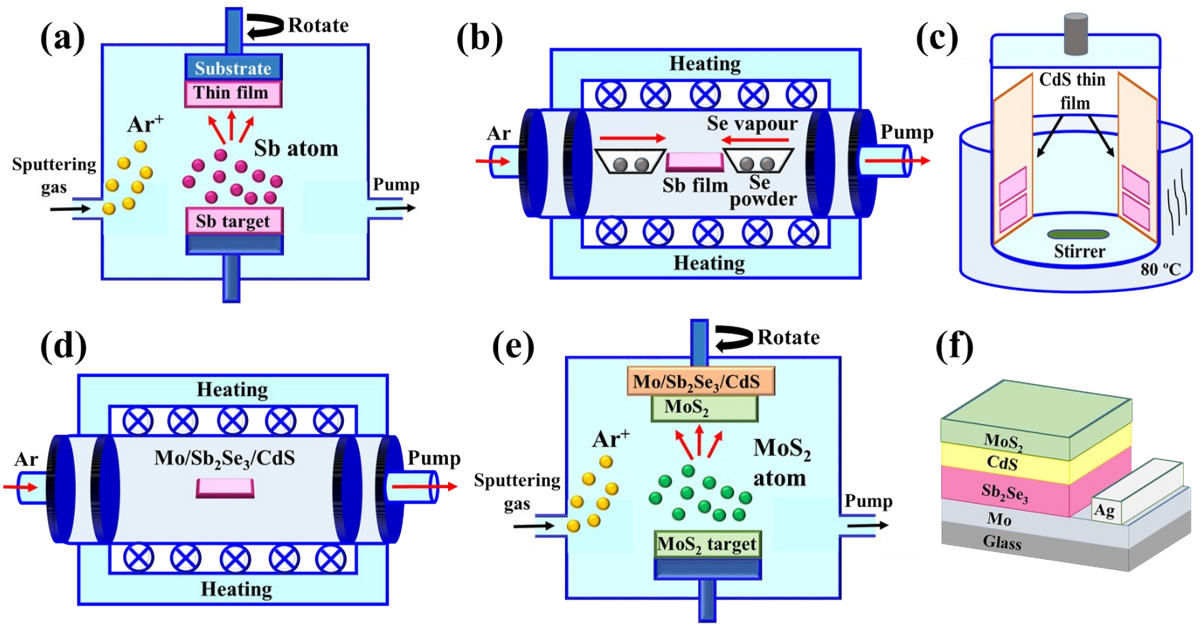

Fig. S1. Schematic illustration of the preparation process of the Sb_2Se_3 thin film photocathode. (a) Sb precursor thin film deposited by RF magnetron sputtering. (b) Sb_2Se_3 thin film obtained by post-selenization heat treatment. (c) CdS buffer layer obtained by CBD method. (d) Post-annealing of the Sb_2Se_3/CdS heterojunction. (e) MoS_2 co-catalyst deposited by RF magnetron sputtering. (f) Schematic configuration of the as-prepared Sb_2Se_3 thin film photocathode.

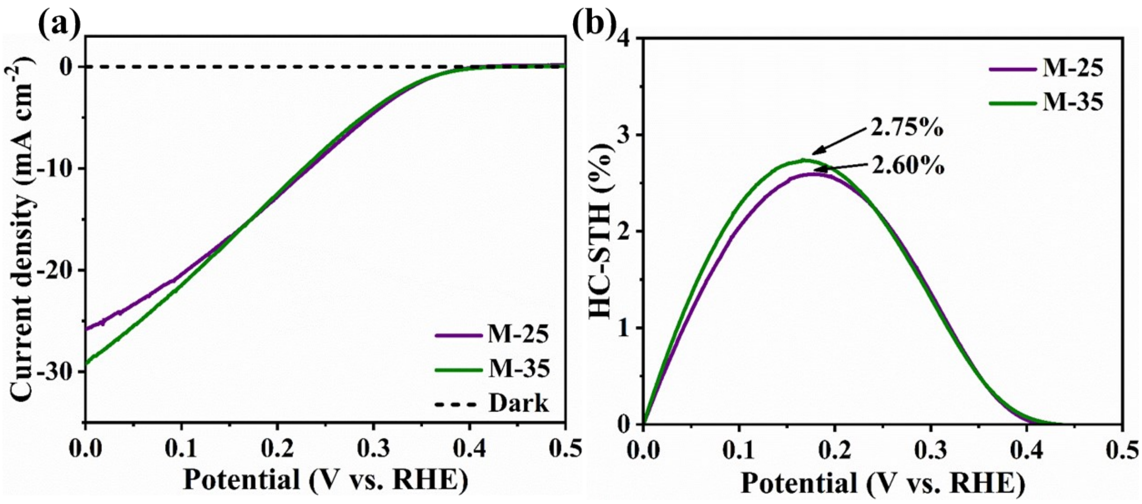

Fig. S2. (a) *J–V* curves of the M-25 and M-35 photocathodes under dark and continuous sunlight illumination, (b) The obtained HC-STH conversion efficiencies.

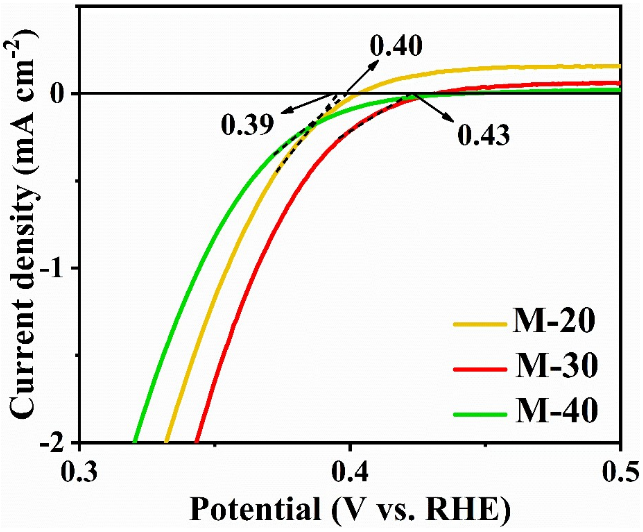

Fig. S3. Enlarged view of the onset potential region of the J-V curves in Figure 1d.

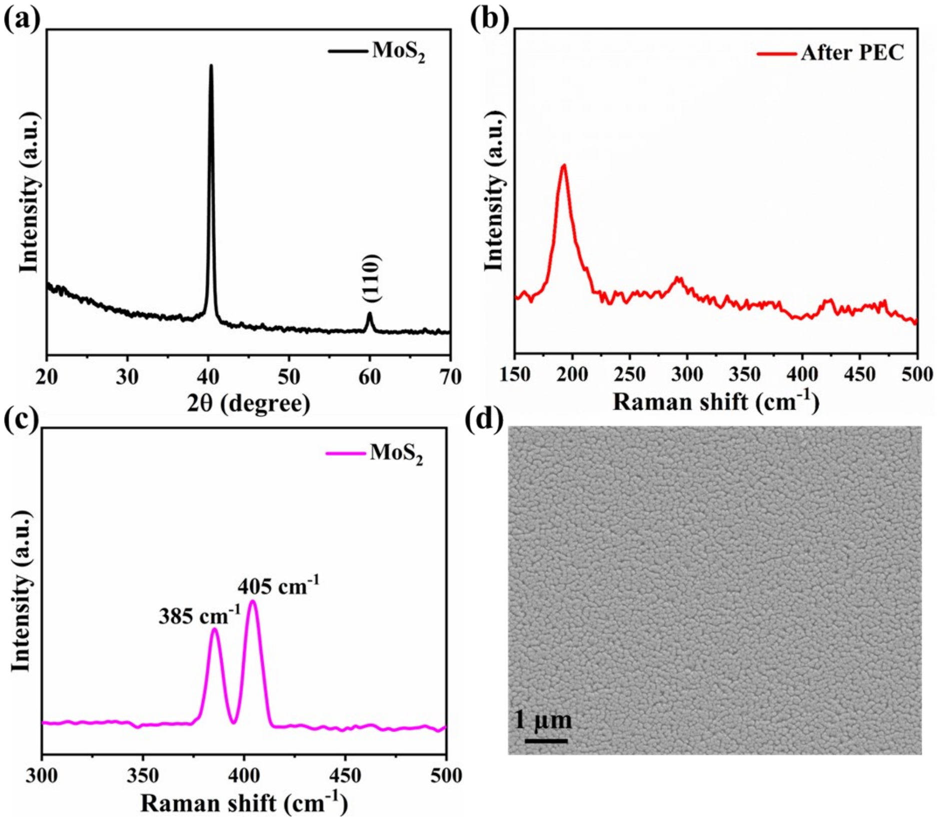

Fig. S4. (a) XRD of pure MoS_2, Raman spectra of (b) M-30 photocathode after PEC measurement, and (c) bare MoS_2 thin film, (d) SEM micrographs of pure MoS_2 thin film.

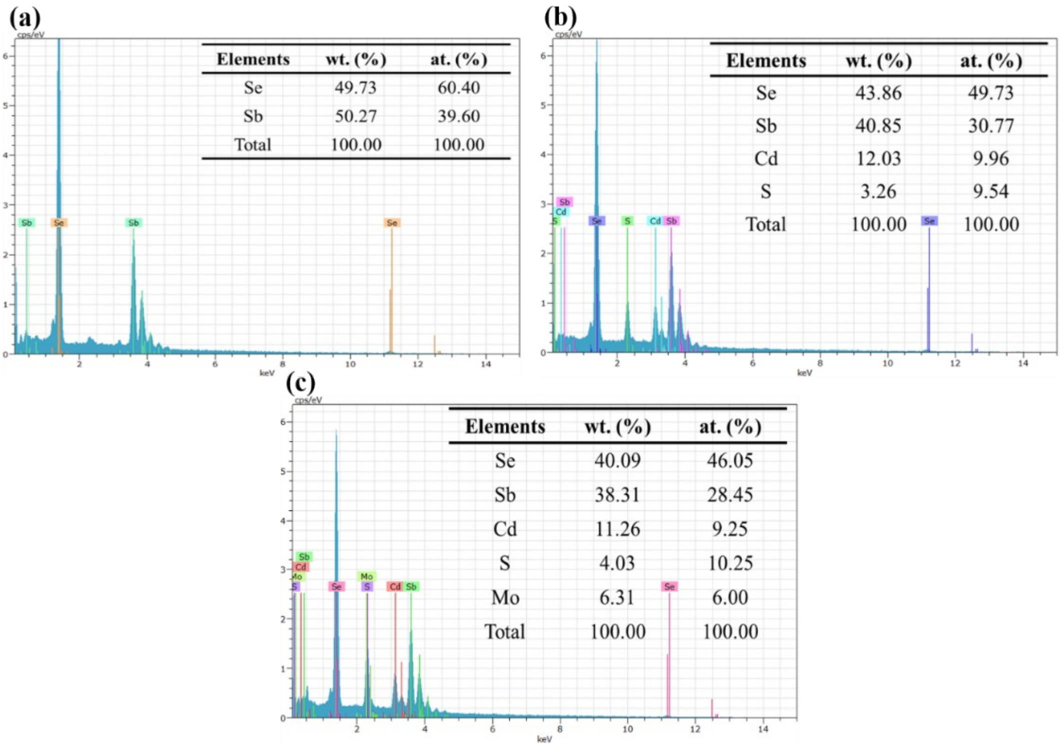

Fig. S5. EDS spectra of (a) Sb_2Se_3, (b) Sb_2Se_3/CdS, (c) Sb_2Se_3/CdS/MoS_2 thin films and their elemental compositions.

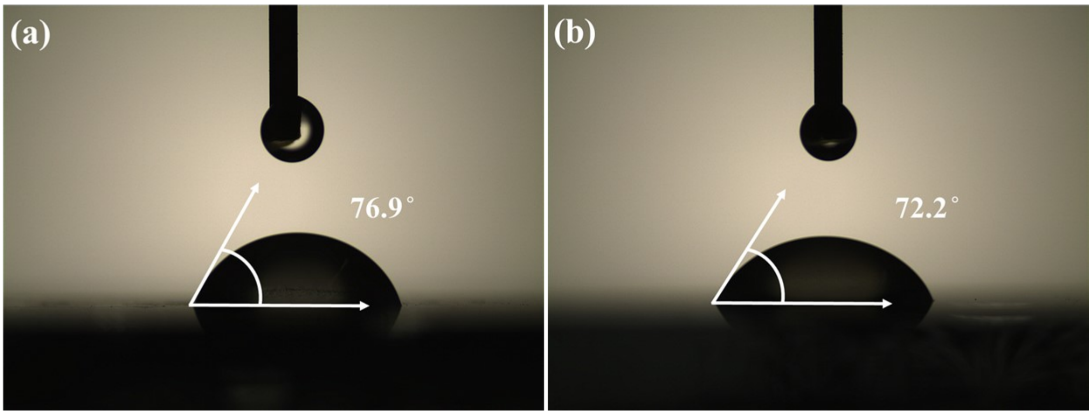

Fig. S6. The measured *CA*s (using H_2SO_4 electrolyte droplet) of (a) M-20 and (b) M-40 samples.

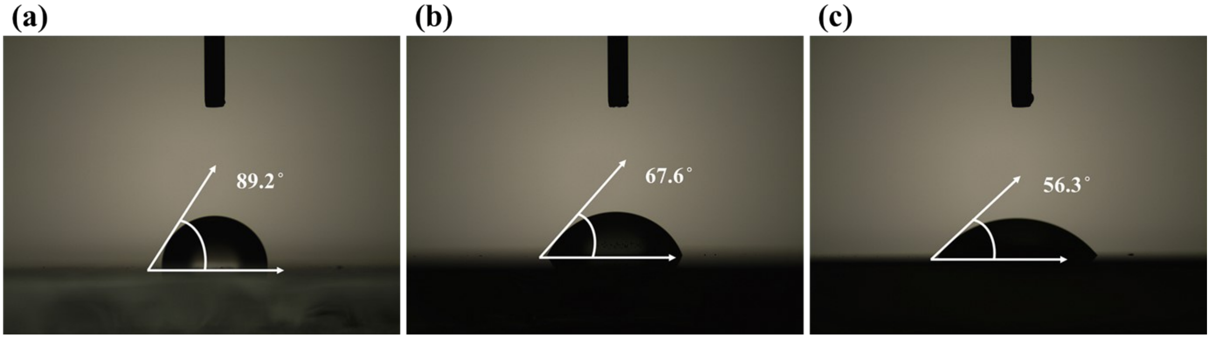

Figure S7. The measured *CA*s (using the glycerol droplet) of (a) Sb_2Se_3, (b) Sb_2Se_3/CdS, and (c) Sb_2Se_3/CdS/MoS_2 samples.

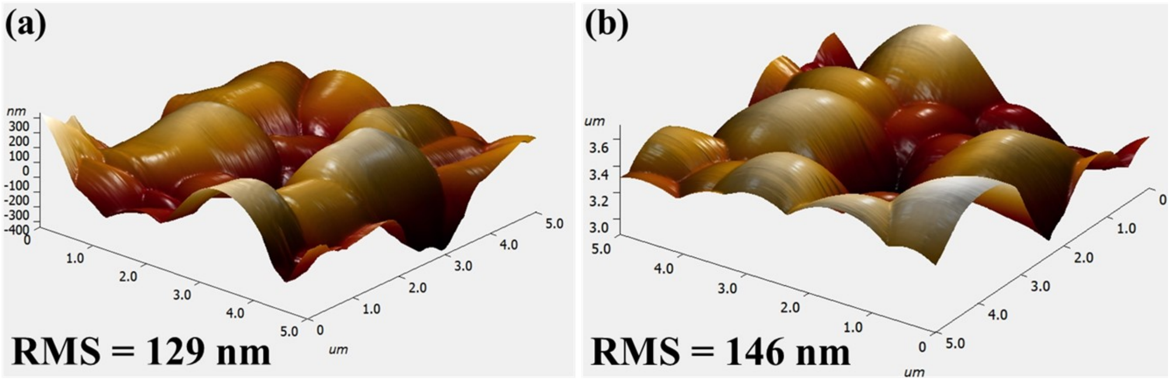

Fig. S8. AFM images of the (a) M-20 and (b) M-40 samples surfaces

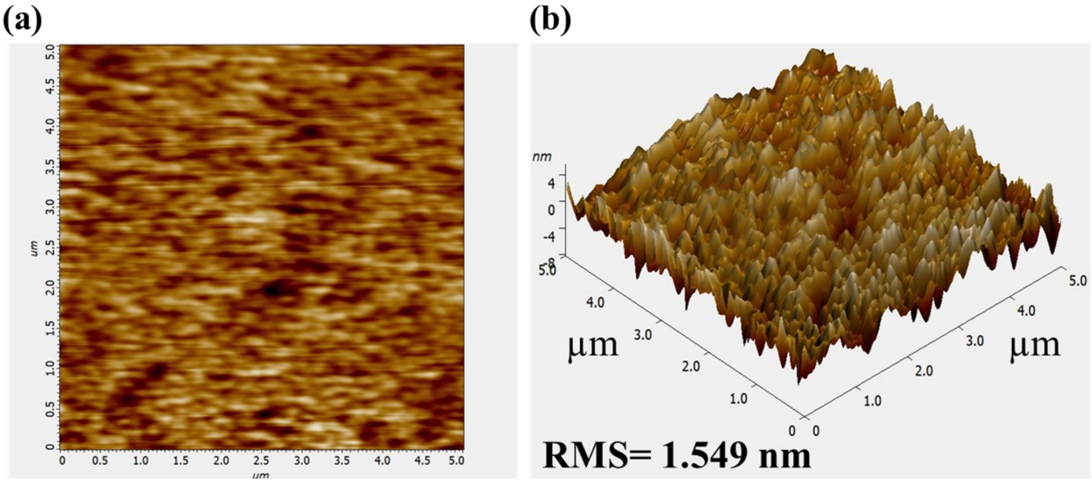

Fig. S9. (a) 2D and (b) 3D AFM images of bare MoS_2 thin film.

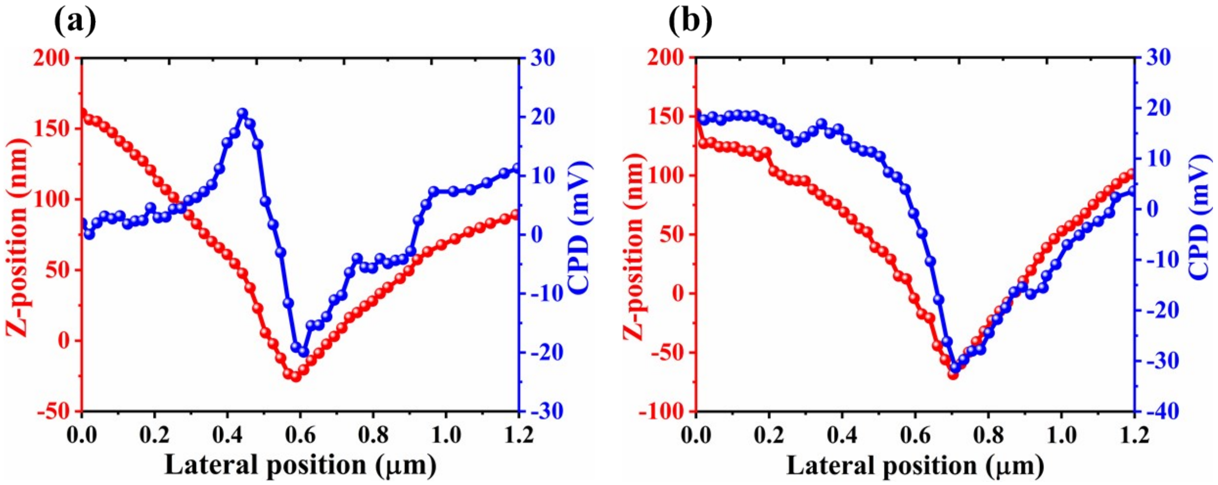

Fig. S10. The topography and potential of (a) M-20, and (b) M-40 photocathodes.

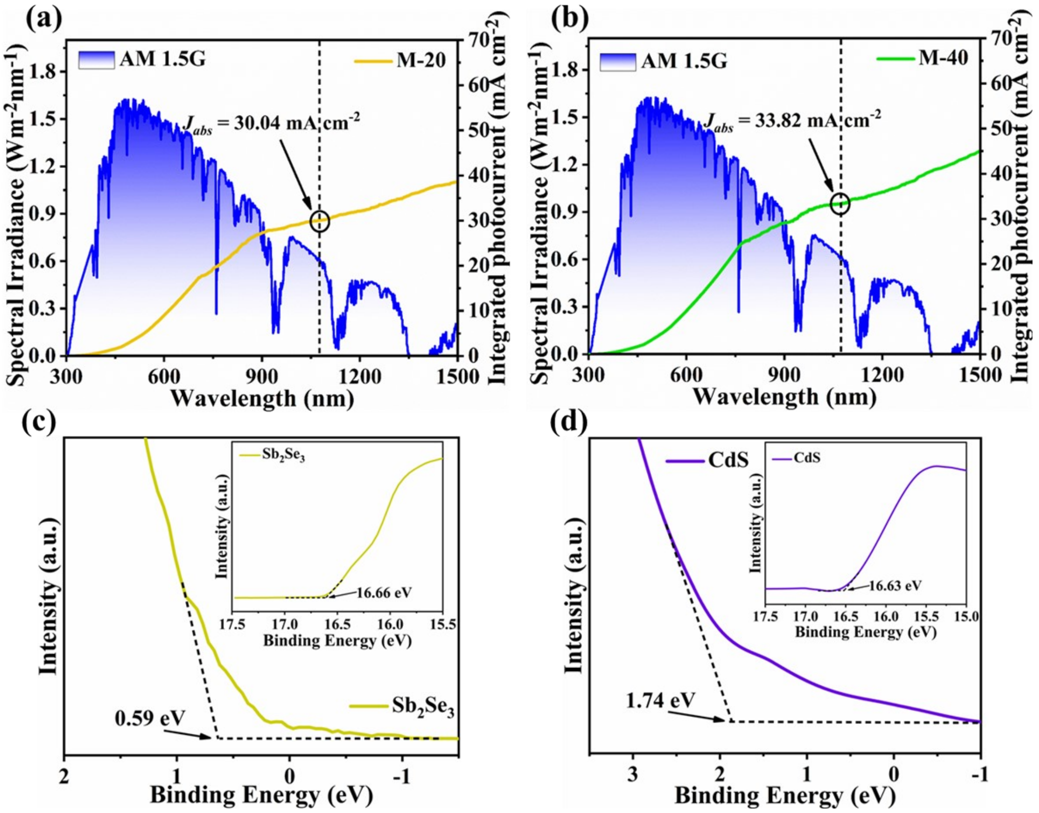

Fig. S11. Energy density flux for the standard (AM 1.5G) solar spectrum and integrated photocurrent density of the (a) M-20, and (b) M-40 samples. (c) UPS characterizations derived V_B positions and SEC edges of (c) Sb_2Se_3, and (d) CdS thin films.

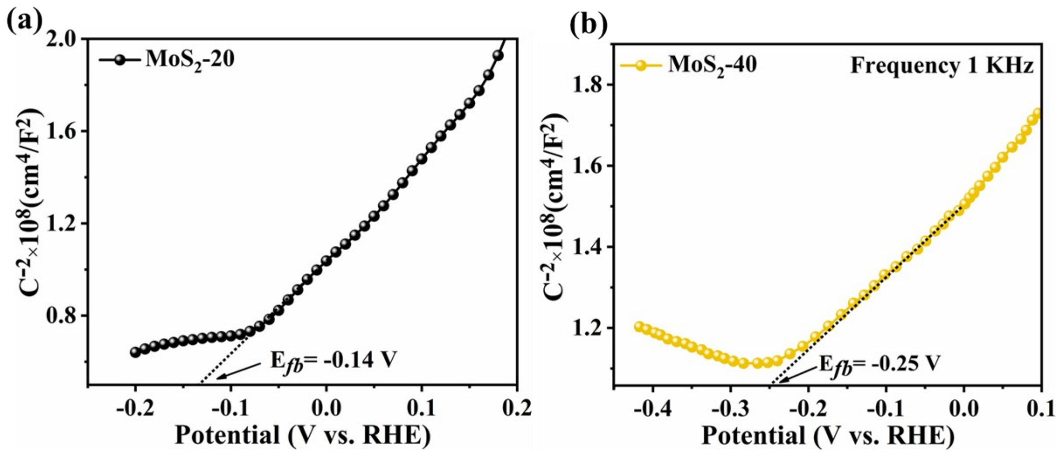

Fig. S12. *M-S* plots of the pure (a) MoS_2-20, and pure (b) MoS_2-40 thin films at a frequency of 1 KHz.

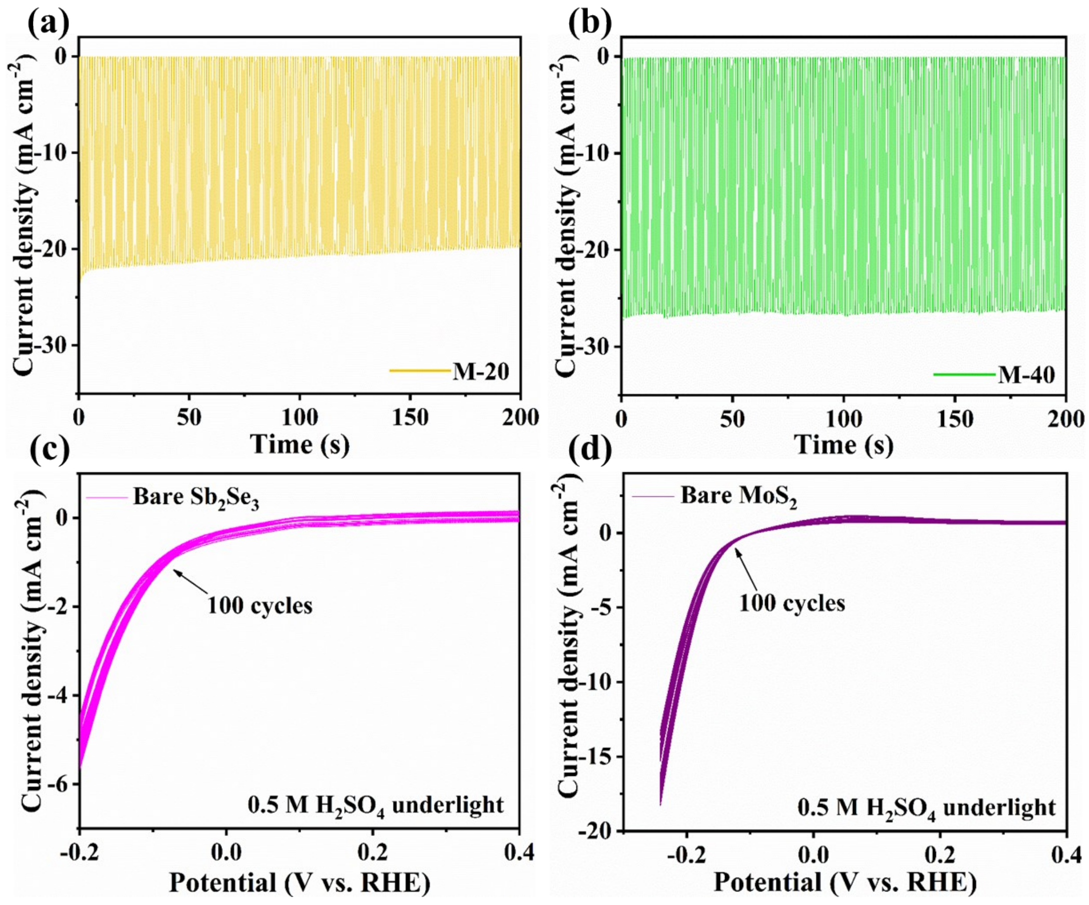

Fig. S13. (a-b) *J-T* curves of the M-20, and M-40 photocathodes at 0 V_{RHE} under AM 1.5G simulated sunlight illumination. Cyclic voltammetry measurements of (c) bare Sb_2Se_3, (d) bare MoS_2 in 0.5 M H_2SO_4 under illumination.

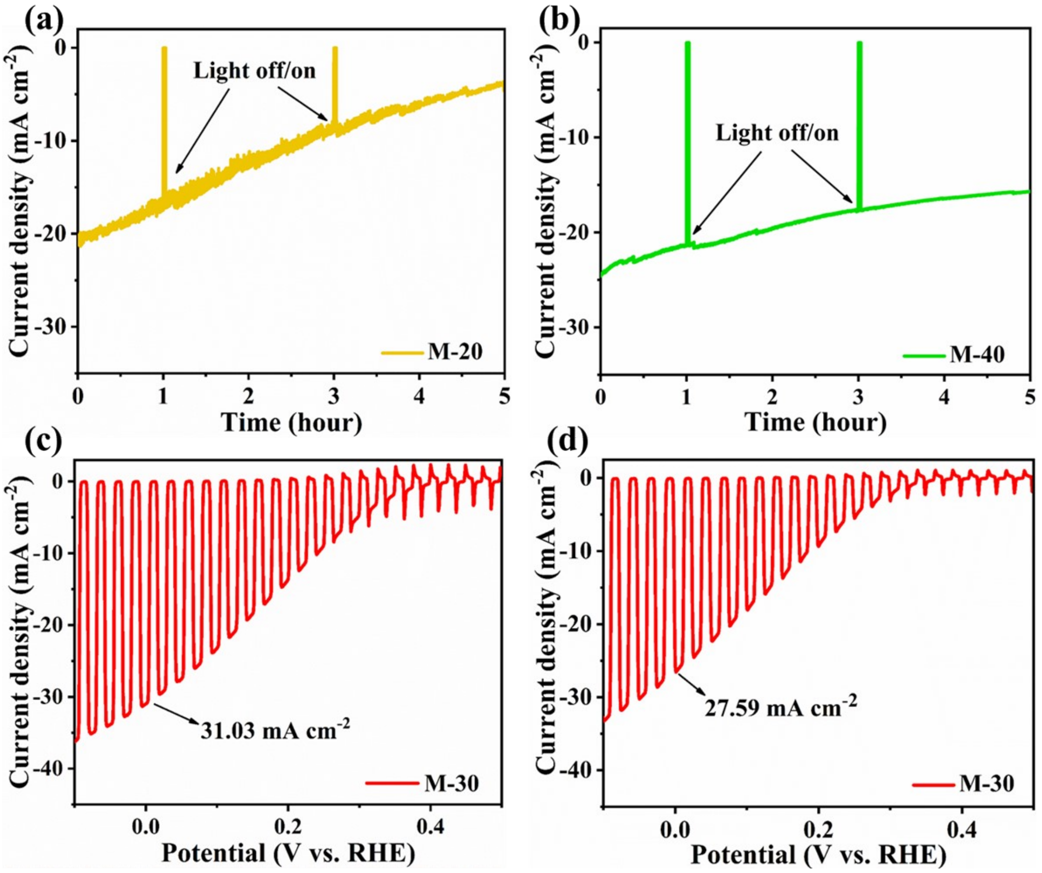

Fig. S14. Photocurrent stability test of the (a) M-20, and (b) M-40 photocathode at 0 V_{RHE} under AM 1.5G sunlight illumination within 5 hours. The chop *LSV* performance of M-30 (c) before and (d) after 5 h stability test.

Supplementary Notes

Note S1

To calculate the theoretical J_{ph} of Sb_2Se_3-based photocathodes using 1.5G AM standard solar light spectrum with wavelength-dependent LHE (light harvesting efficiency), the following formula were applied (Figure S8). [7, 8]

$$J_{abs} = \int_{300}^{\lambda_e} \frac{\lambda}{1240} . N_{ph}(\lambda) \times LHE(\lambda) d\lambda \quad (1)$$

$$LHE = 1 - 10^{-A(\lambda)} \quad (2)$$

where J_{abs} is theoretical photocurrent density, λ is wavelenth, λ_e is absorption cut-off wavelenth related to band-gap, $N_{ph}(\lambda)$ phototon flux, and $A(\lambda)$ is absorbance related to wavelenth.

References

1 H. Zhou, M. Feng, K. Song, B. Liao, Y. Wang, R. Liu, X. Gong, D. Zhang, L. Cao and S. Chen, *Nanoscale*, 2019, **11**, 22871-22879.

2 H. Zhou, M. Feng, M. Feng, X. Gong, D. Zhang, Y. Zhou and S. Chen, *Appl. Phys. Lett.*, 2020, **116**, 113902.

3 M. Wang, S. Wang, Q. Zhang, S. Pan, Y. Zhao and X. Zhang, *Sol. RRL*, 2022, **6**, 2100798.

4 J. Tan, W. Yang, H. Lee, J. Park, K. Kim, O. S. Hutter, L. J. Phillips, S. Shim, J. Yun, Y. Park and J. Lee, *Appl. Catal., B*, 2021, **286**, 119890.

5 W. Yang, J. H. Kim, O. S. Hutter, L. J. Phillips, J. Tan, J. Park, H. Lee, J. D. Major, J.S. Lee and J. Moon, *Nat. Commun.*, 2020, **11**, 861.

6 J. Park, W. Yang, Y. Oh, J. Tan, Lee, H., R. Boppella, and J. Moon, *ACS Energy Lett.*, 2019, **4**, 517-526.

7 G. Liang, T. Liu, M. Ishaq, Z. Chen, R. Tang, Z. Zheng, Z. Su, P. Fan, X. Zhang and S. Chen, *Chem. Eng. J.,* 2022, **431**, 133359.

8 T. Zhou, S. Chen, J. Wang, Y. Zhang, J. Li, J. Bai and B. Zhou, *Chem. Eng. J.,* 2021, **403**, 126350.
